# Huc-MSC-derived exosomes modified with the targeting peptide of aHSCs for liver fibrosis therapy

**DOI:** 10.1186/s12951-022-01636-x

**Published:** 2022-10-01

**Authors:** Yan Lin, Mengchao Yan, Zhongtian Bai, Ye Xie, Longfei Ren, Jiayun Wei, Dan Zhu, Haiping Wang, Yonggang Liu, Junqian Luo, Xun Li

**Affiliations:** 1 The First Clinical Medical College of Lanzhou University, Lanzhou, China; 2 Department of General Surgery, The First Hospital of Lanzhou University, Lanzhou, China; 3 Key Laboratory of Biotherapy and Regenerative Medicine of Gansu Province, Lanzhou, China; 4 Guangzhou Key Laboratory for Surface Chemistry of Energy Materials, New Energy Research Institute, School of Environment and Energy, South China University of Technology, Guangzhou Higher Education Mega Centre, Guangzhou, China; 5 Jinzhong Hospital Affiliated to Shanxi Medical University, Jinzhong, China

**Keywords:** Liver fibrosis, aHSCs, Huc-MSCs, Targeting peptide, Exosomes

## Abstract

**Background:**

Effective therapeutics to stop or reverse liver fibrosis have not emerged, because these potential agents cannot specifically target activated hepatic stellate cells (aHSCs) or are frequently toxic to parenchymal cells. Human umbilical cord mesenchymal stem cell (Huc-MSC)-derived exosomes show promise in nanomedicine for the treatment of liver fibrosis. However, systemic injection showed that unmodified exosomes were mainly taken up by the mononuclear phagocyte system. The discovery of ligands that selectively bind to a specific target plays a crucial role in clinically relevant diagnostics and therapeutics. Herein, we aimed to identify the targeting peptide of aHSCs by screening a phage-displayed peptide library, and modify Huc-MSC-derived exosomes with the targeting peptide.

**Results:**

In this study, we screened a phage-displayed peptide library by biopanning for peptides preferentially bound to HSC-T6 cells. The identified peptide, HSTP1, also exhibited better targeting ability to aHSCs in pathological sections of fibrotic liver tissues. Then, HSTP1 was fused with exosomal enriched membrane protein (Lamp2b) and was displayed on the surface of exosomes through genetic engineering technology. The engineered exosomes (HSTP1-Exos) could be more efficiently internalized by HSC-T6 cells and outperformed both unmodified exosomes (Blank-Exos) and Lamp2b protein overexpressed exosomes (Lamp2b + Exos) in enhancing the ability of exosomes to promote HSC-T6 reversion to a quiescent phenotype. In vivo results showed HSTP1-Exos could specifically target to the aHSC region after intravenous administration, as demonstrated by coimmunofluorescence with the typical aHSCs marker α-SMA, and enhance the therapeutic effect on liver fibrosis.

**Conclusion:**

These results suggest that HSTP1 is a reliable targeting peptide that can specifically bind to aHSCs and that HSTP1-modified exosomes realize the precise treatment for aHSCs in complex liver tissue. We provide a novel strategy for clinical liver fibrosis therapy.

**Graphical Abstract:**

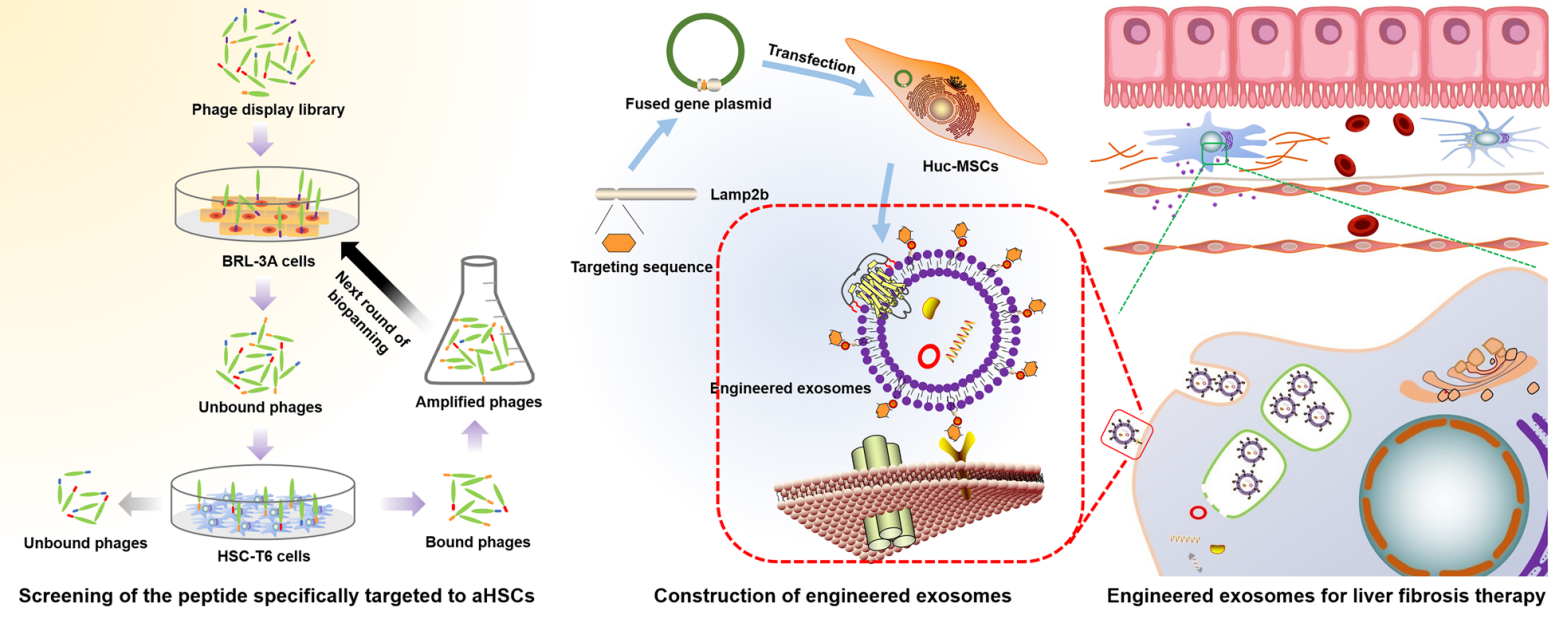

## Introduction

Chronic liver disease is responsible for approximately 2 million global deaths per year, as well as a large economic burden and increased healthcare utilization [1]. Hepatic fibrosis initiated as a result of chronic liver injury due to virus infection, alcoholic liver disease, nonalcoholic fatty liver disease, drug toxicity, metabolic disorders and autoimmune disease, is believed to initiate the deterioration of liver architecture [2, 3]. Liver fibrosis is characterized by excessive accumulation of extracellular matrix (ECM), and aHSCs are the main cell population for the ECM [4, 5]. Under physiological conditions, HSCs reside in the space of Disse between the liver sinusoidal endothelial cells and hepatocytes in the quiescent state [6]. Quiescent hepatic stellate cells (qHSCs) are identified with lipid-rich vacuoles that store vitamin A in the form of retinyl ester, and play a critical role in the regulation of immune modulation, regeneration and lipid metabolism [7]. When the liver is damaged, qHSCs are activated and differentiate to a myofibroblast-like phenotype, and lose their lipid droplets (LDs) and their expression of α-smooth muscle actin (α-SMA) and type I collagen (Col I) [8, 9]. The good plasticity of HSCs lies in the fact that the activated phenotype can undergo morphologic and biochemical reversal to a quiescent state [8, 10]. Thus, aHSCs have become attractive therapeutic targets.

Mesenchymal stem cells(MSCs) are multipotent stem cells with widespread relevance in the field of regenerative medicine owing to their tissue repair and immune regulation [11]. Recently, accumulating studies have evaluated whether exogenous MSCs can ameliorate HSC activation to reduce fibrosis and improve liver function [12, 13]. Despite their beneficial effects for treating liver diseases, the application of MSCs is limited by iatrogenic tumor formation and low engraftment in vivo. Moreover, cellular rejection, infusion toxicity and induced occlusion in the microvasculature inhibit the clinical application of MSCs in current regenerative medicine [14, 15]. The therapeutic potential of stem cells has been attributed to exosome-based paracrine factors [16]. MSC-derived exosomes retain similar molecular cargo similar to that of parent MSCs, which can also significantly decrease the activation of HSCs [17, 18]. Exosomes are less complex and smaller than their parent cells, and they present no risk of tumor formation. A lower content of membrane-bound protein, confers less immunogenicity in exosomes [15]. Exosomes may be a novel cell-free therapy.

However, the efficiency is compromised, as the spleen with mononuclear phagocytes is also the target organ for unmodified (or native) exosomes in addition to the liver [19, 20]. This phenomenon results in the accumulation of exosomes in nonspecific organs with a waste of biological agents. HSCs are mesenchymal cells and comprise approximately 15% of total resident cells in normal human liver and only 5–8% of total rat liver cells. In mouse liver, HSCs account for 8–10% of total liver cells, and expand to15% in fibrogenic injury liver [21, 22]. Hepatic sinusoids will form pathological changes, termed ‘capillarization’ in liver fibrosis and cirrhosis [23]. For these reasons, effective therapeutic concentrations cannot be enriched around aHSCs.

To solve the above problems, we intended to engineer exosomal membrane proteins, which have attracted the attention of many scholars in this area [24]. In this work, we aimed to identify the targeting peptide of aHSCs by screening a phage-displayed peptide library, and then fusing the targeting peptide with Lamp2b. We expected to achieve breakthroughs for liver fibrosis therapy.

## Results

### Phage enrichment analysis and identification of positive phage clones

**Fig. 1:**
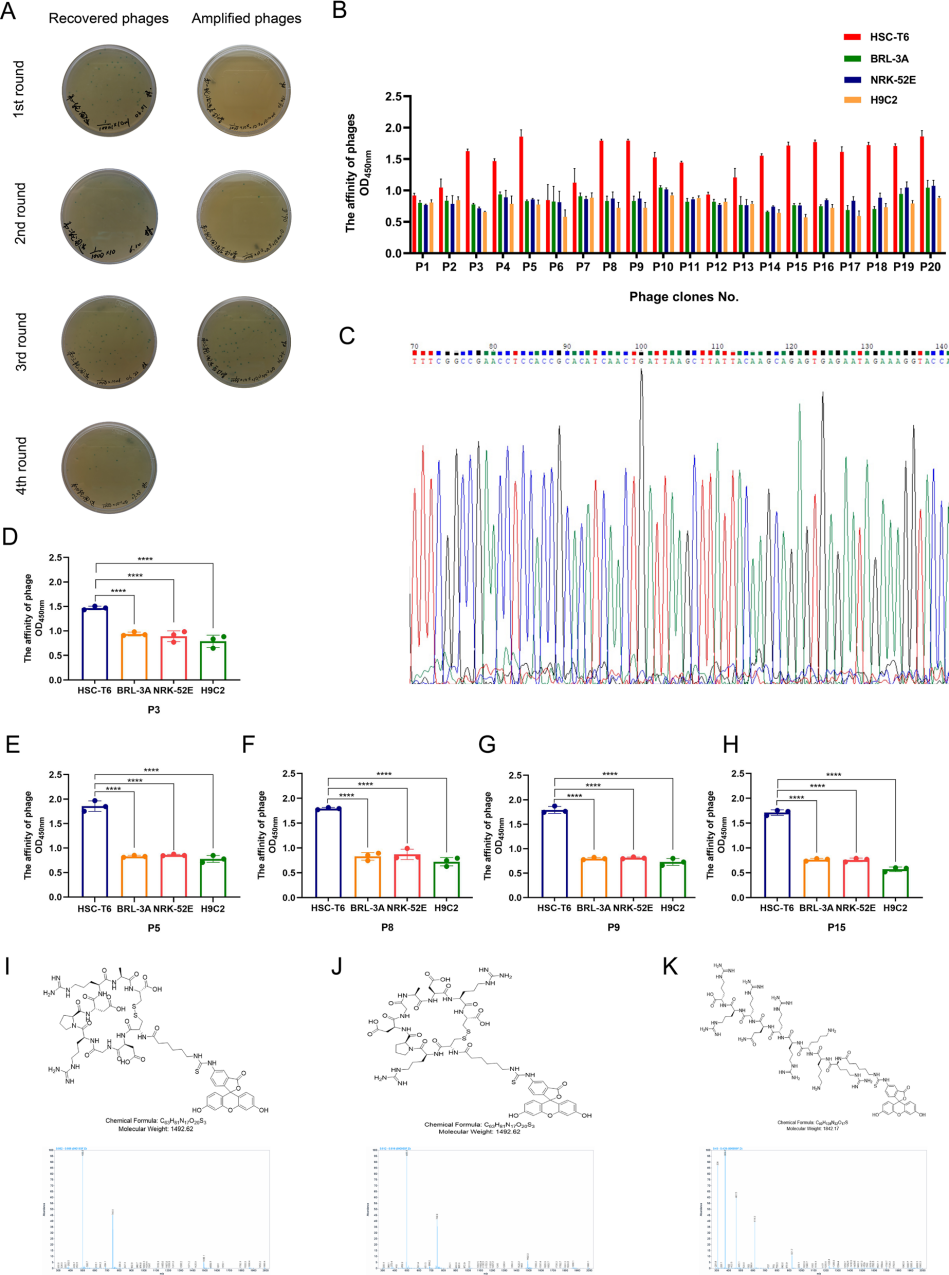
Screening of phages and identification of positive phage clones. **A** The titers of the recovered and amplified phages from each round. The blue plaques formed on agar plates containing tetracycline were used to calculated the phages. **B** Elisa results for 20 phage clones binding to HSC-T6, BRL-3A, NRK-52E, and H9C2 cells. **C** Following 4 rounds of phage display biopanning, amino acid sequence with highest frequencies was identified by DNA sequencing. The nucleic acid sequences were analyzed by Chromas. **D**–**H** Comparison of phage cell-binding Elisa results for the affinity of individual phage clones with same sequence (CDGRPDRAC) to HSC-T6, BRL-3A, NRK-52E, and H9C2 cells. **I**–**K** Molecular structure and mass spectrogram of the HSTP1, rcHSTP1, and TAT. *P < 0.05, **P < 0.01, ***P < 0.001, ****P < 0.0001

The bound phages were rescued and amplified in *E*. coli 2738, and both were tittered in each round (Fig. 1A). The input titer of the phages was 2 × 10^11^ pfu for each round, and the recovery efficiency in the third round was approximately 46-fold that in the first round (Table 1). Twenty plaques were randomly selected, and the affinity of all the clones with HSC-T6 cells was better than that with BRL-3A, NRK-52E, and H9C2 cells (Fig. 1B). We found that the phages P3, P5, P8, P9, and P15 had the same sequence in DNA sequencing (Fig. 1C). Moreover, they all indicated a higher affinity for HSC-T6 by using cellular ELISAs (Fig. 1–H). Due to its highest frequency of enrichment through screens, was chosen for further characterization and termed HSTP1. HSTP1, rcHSTP1, and Tat were synthesized and commercially purified, and labeled with FITC (Fig. 1–K).

**Table 1:** Recovery efficiency of phage display biopanning increases round by round

Round of screen	Input phage(pfu)	Output phage(pfu)	Yield
1	2 × 10^11^	5.1 × 10^5^	2.6 × 10^–6^
2	2 × 10^11^	2.6 × 10^6^	1.3 × 10^–5^
3	2 × 10^11^	4.3 × 10^6^	2.2 × 10^–5^
4	2 × 10^11^	2.3 × 10^7^	1.2 × 10^–4^

### Assessing the targeting affinity of the peptide

**Fig. 2:**
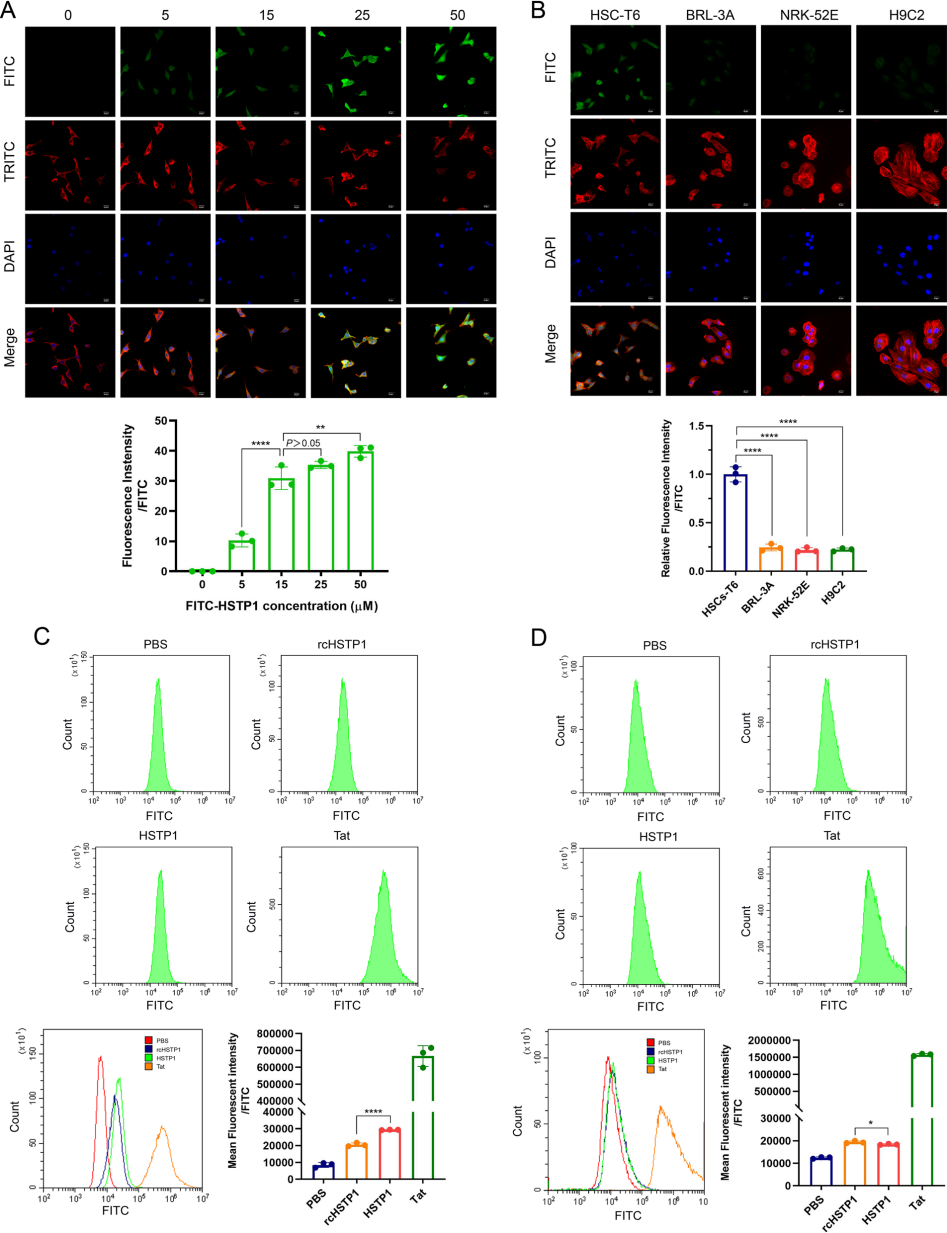
Assessing the targeting affinity of the HSTP1 to aHSCs by immunocytofluorescence and flow cytometry. **A** Confocal microscopic images analysis of the binding affinity of the HSTP1 in different concentrations. **B** Confocal microscopic images analysis of the binding specificity of the HSTP1 to HSC-T6 cells. **C**, **D** Compared with negative control group (rcHSTP1), identified HSTP1 demonstrated a high affinity to HSC-T6 cells and had a superiority of amino acid sequence by flow cytometry. The bar graph depicts the mean fluorescence intensity of FITC. *P < 0.05, **P < 0.01, ***P < 0.001, ****P < 0.0001. Scale bar: 20 µm

**Fig. 3:**
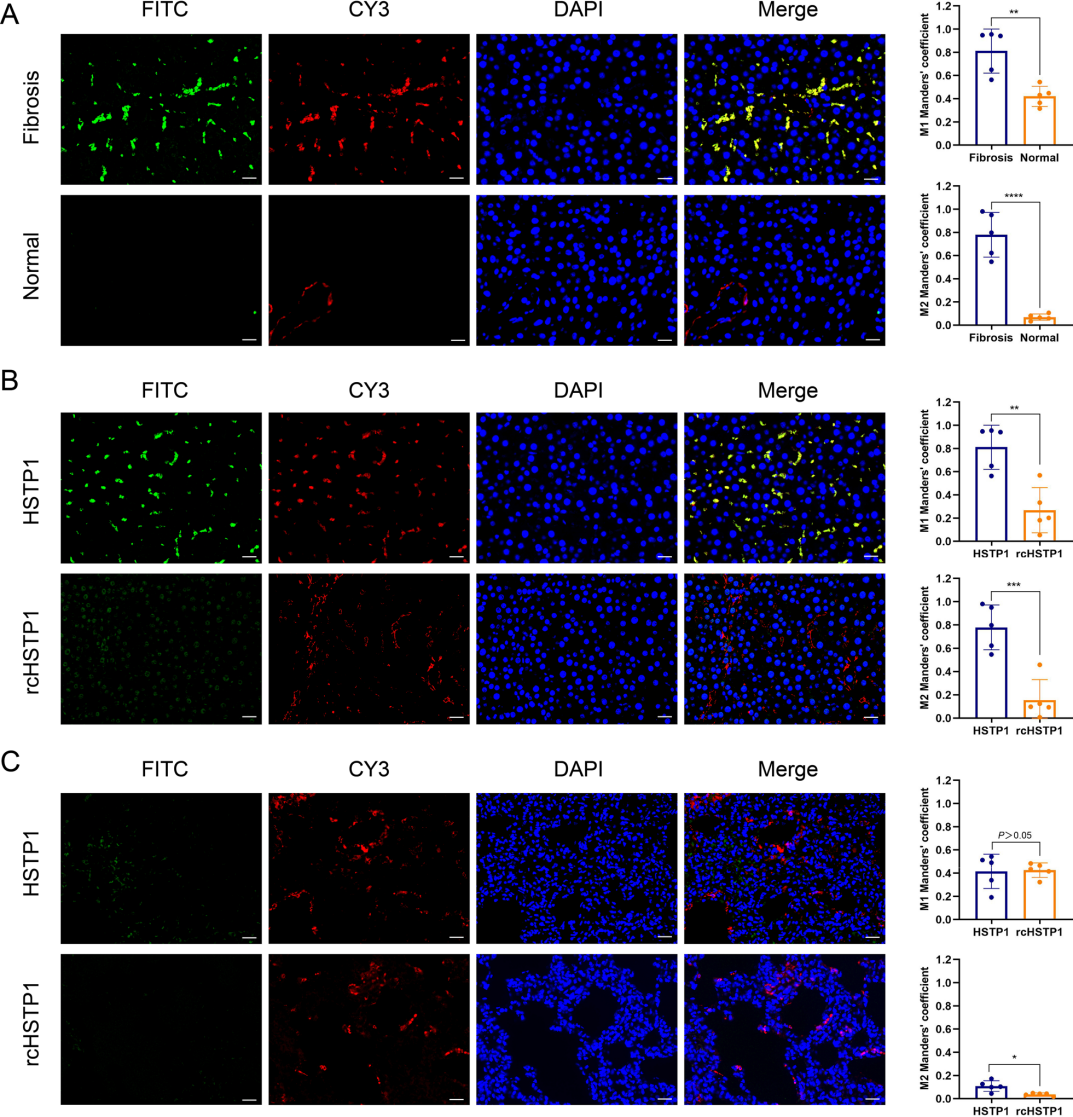
Assessing the specificity of the HSTP1 to aHSCs by immunofluorescence histochemistry. **A** aHSCs and HSTP1 co-immunofluorescence in fibrotic and normal liver tissues. The aHSCs were stained with anti-α-SMA antibody (red), FITC-labeled HSTP1 (green), nuclei were visualized by counterstaining with DAPI (blue), and images were merged. **B** Co-immunofluorescence for HSTP1, rcHSTP1 (green) and α-SMA (red) in fibrotic liver tissues. **C** Co-immunofluorescence for HSTP1, rcHSTP1 (green) and α-SMA (red) in lung tissues. The bar graphs depict the Manders’ coefficients M1 and M2 in colocalization. Scale bar: 50 µm. *P < 0.05, **P < 0.01, ***P < 0.001, ****P < 0.0001

FITC-HSTP1 was incubated with HSC-T6 cells, followed by confocal microscopy analysis. The fluorescence signal gradually intensified with increasing concentrations. We found that the signal was already very obvious when the concentration was at 15 µM (Fig. 2A). To test the specificity of HSTP1, we incubated HSC-T6, BRL-3A, NRK-52E, and H9C2 cells with 15 µM FITC-HSTP1, and observed them by fluorescence confocal microscopy. There was a very obvious fluorescent signal in HSC-T6 cells, but relatively low fluorescent signals were observed in the other three cell lines treated with FITC-HSTP1 (Fig. 2B). We comparatively analyzed the affinity of HSTP1 and a scrambled sequence of HSTP1 (rcHSTP1) to HSC-T6 and BRL-3A cells via flow cytometry. Tat peptide was used as the positive control. We found that the average fluorescence intensity was 29,320.6 ± 150.5 for the HSC-T6 cells incubated with FITC-HSTP1, which was significantly higher than 20,523.7 ± 1049.4 for the HSC-T6 cells incubated with rcHSTP1 (Fig. 2C). Moreover, the average fluorescence intensity was 18,400.9 ± 240.1 for the BRL-3A cells incubated with FITC-HSTP1, which was significantly lower than the 19,408.6 ± 495.9 for the BRL-3A cells incubated with rcHSTP1 (Fig. 2D). We further examined the targeting of HSTP1 to aHSCs in pathological tissues. We found that HSTP1 could specifically bind to aHSCs in paraffin sections of liver fibrosis, as demonstrated by co-immunofluorescence with the typical aHSCs marker α-SMA. The colocalization of peptides and α-SMA staining were determined by measuring the Manders’ coefficients M1 and M2. An obvious fluorescent signal in fibrotic liver tissues but relatively weak fluorescent signals were observed in normal liver tissues. In normal liver, α-SMA labeled vascular smooth muscle cells. However, there was little colocalization of peptides and α-SMA staining (Fig. 3A). In comparison to HSTP1 peptide, there was a very weak fluorescent signal in all cells of paraffin sections of liver fibrosis when incubated with rcHSTP1. The M1 and M2 coefficients of the rcHSTP1 were 0.27 (*P* < 0.01) and 0.16 (*P* < 0.001) (Fig. 3B). Meanwhile, we labeled fibroblasts in lung tissue with the α-SMA staining. Both HSTP1 and rcHSTP1 displayed very low fluorescent signal. The average M1 and M2 coefficients of two group were less than 0.6 (Fig. 3C). These results support the idea that HSTP1 is a targeting peptide for aHSCs.

### Identification and modification of huc-MSCs

**Fig. 4:**
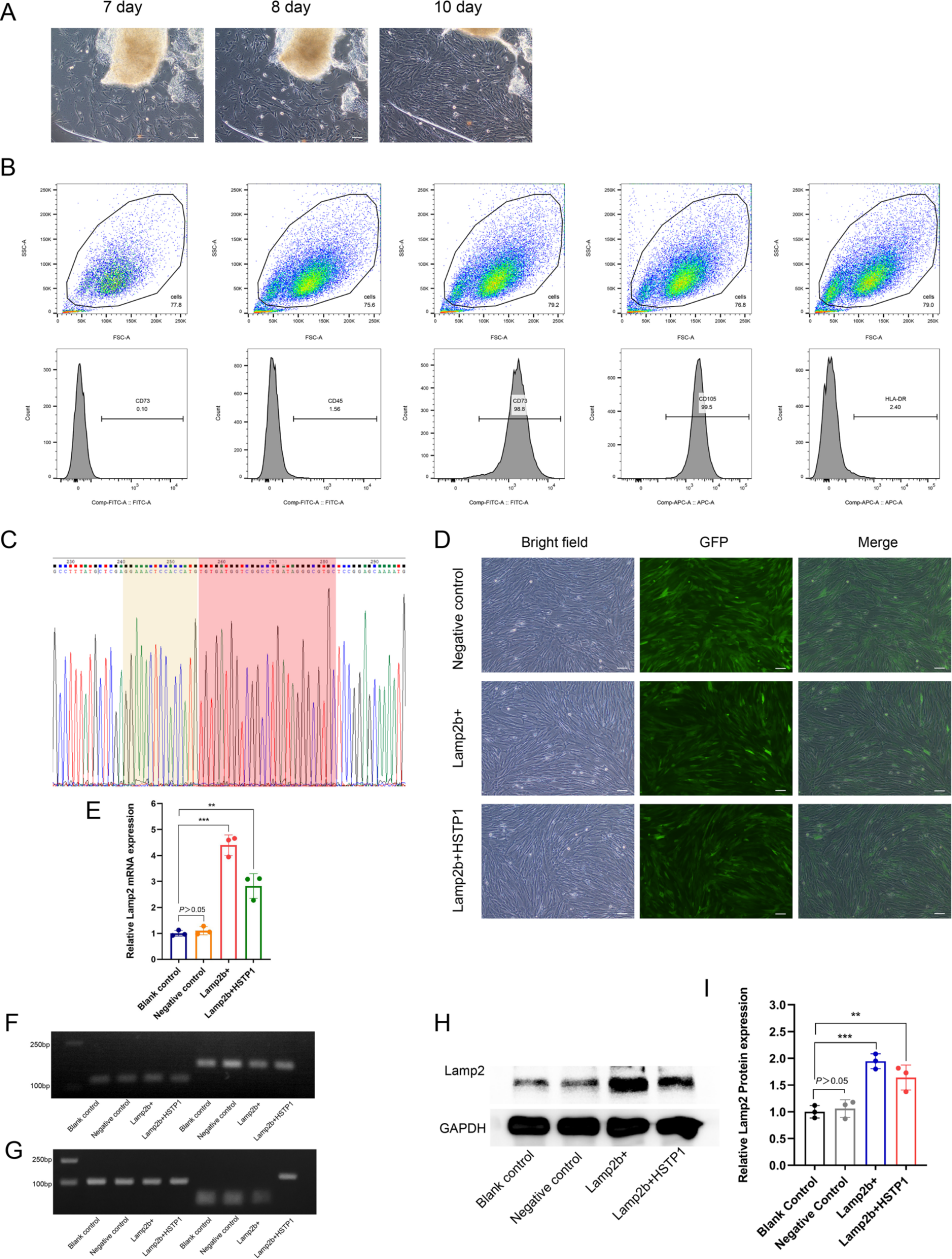
Isolation, identification, and modification of huc-MSCs. **A** Inverted phase contrast microscopy images of cells morphological observation of huc-MSCs. Scale bar: 100 µm. **B** Identification of huc-MSCs with CD45, CD73, CD105, and HLA-DR. **C** Sequencing results of fused gene of Lamp2b and HSTP1 constructed in plasmid vectors. Sequence of yellow shading was GNSTM, sequence of red shading was HSTP1. **D** Fluorescence microscope images of infection efficiency of empty vector lentivirus (negative control), Lamp2b gene overexpression lentivirus (Lamp2b +), and fused gene overexpression lentivirus (Lamp2b + HSTP1) in huc-MSCs. **E** RT-PCR results of Lamp2 mRNA expression in Blank control, Negative control, Lamp2b + , and Lamp2b + HSTP1 with the universal primer (forward 5’-AACCCCAATACAACTCACTCC-3’, reverse 5’-GCCATTAACCAAATACATGCTG-3’). **F** Agarose gel electrophoresis of RT-PCR products with the universal primer. G Agarose gel electrophoresis of RT-PCR products with the specific primer (forward 5’-AGGAAACTCCACCATGTGTGATG-3’, reverse 5’-GCTTCCATTATATGTCACAGTGCC-3’). H Western blot and relative protein expression qualification for the Lamp2 in huc-MSCs. *P < 0.05, **P < 0.01, ***P < 0.001, ****P < 0.0001

Cell morphological features of huc-MSCs were observed using inverted phase contrast microscopy. Migrated cells were observed around umbilical cord tissue blocks in approximately 7 days, and the cells were fusiform or polygonal. When cultured for 10 days, the cells were arranged densely, grew in parallel or in a whirlpool, pattern and were similar to fibroblasts (Fig. 4A). Cells trended toward homogenization and stability after being subcultured up to the third passage. Importantly, the immunophenotype of these cells was detected by flow cytometry and revealed that these cells were positive for CD73 and CD105, but negative for CD45 and HLA-DR (Fig. 4B). Taken together, these findings indicated that we had efficiently generated huc-MSCs. We comparatively analyzed the sequencing results of the constructed plasmid with the sequence of HSTP1. We found that the fusion gene of Lamp2b + HSTP1 was constructed successfully in vectors. Moreover, we designed the GNSTM motif inserted at the *N*-terminal of the inserted sequence of HSTP1 (Fig. 4C). The day before transfection, huc-MSCs were plated at a density of 1 × 10^4^ cells in 6-well plates so that they were 20–30% confluent on the day of transfection. After 11 days, obvious green fluorescence signals were observed in the cells using fluorescence microscope. The infection efficiency of the fused gene (Lamp2b + HSTP1) overexpression lentivirus, Lamp2b gene overexpression lentivirus and empty vector lentivirus was 80% to 90% (Fig. 4D). To determine whether HSTP1 was fused with Lamp2b, we performed RT-PCR, agarose gel electrophoresis of RT-PCR products and Western blotting. We found that target genes were efficiently amplified in all cells using universal primers of Lamp2b (Fig. 4E, F). However, only the fused gene was amplified using specific primers of Lamp2b + HSTP1 (Fig. 4G). Lamp2b and Lamp2b + HSTP1 proteins expression was upregulation (Fig. 4H, I). These data verified that HSTP1 was successfully fused with Lamp2b.

### Characterization of exosomes

**Fig. 5:**
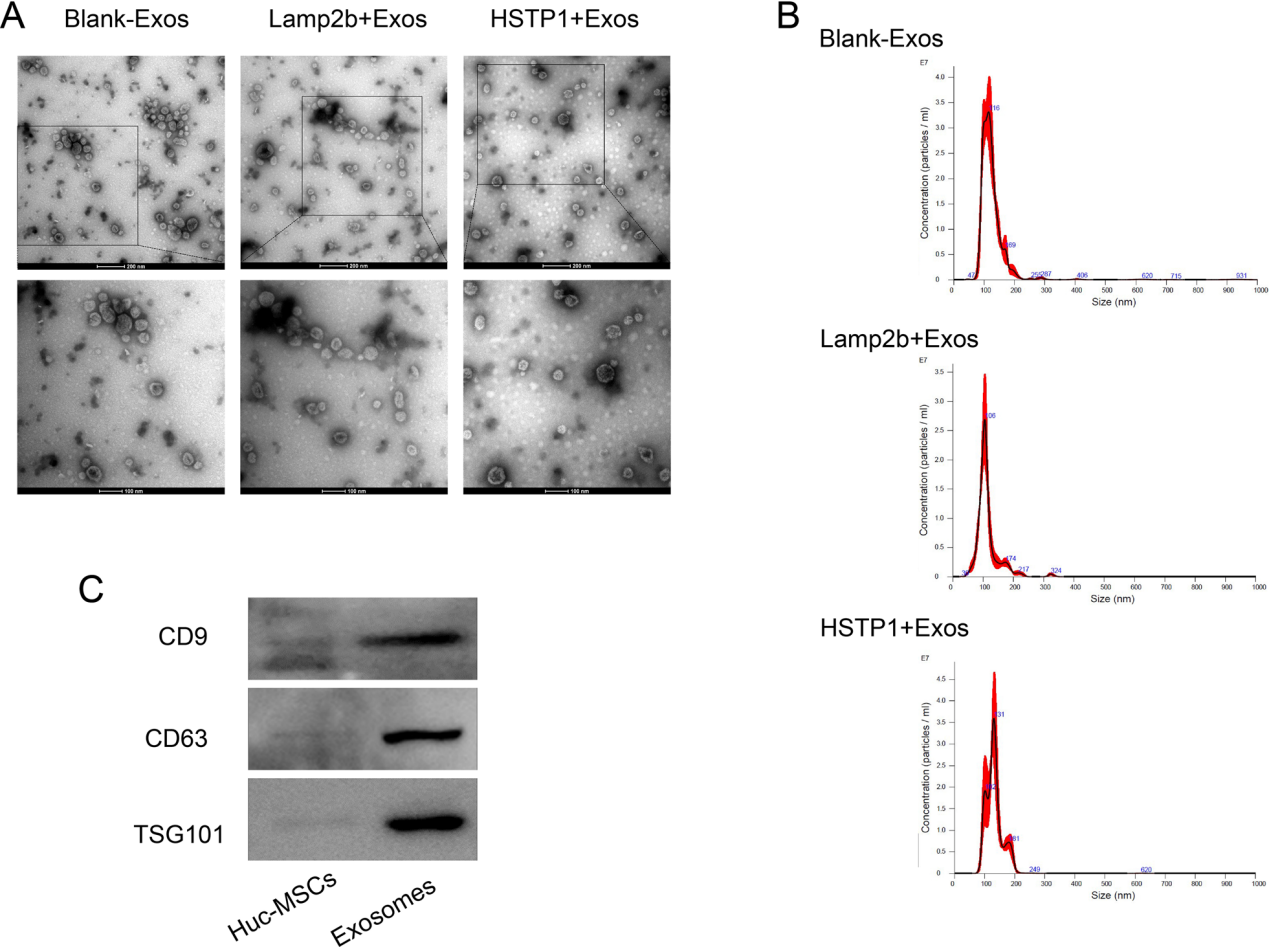
Identification of exosomes. **A** Size distribution of Blank-Exos, Lamp2b-Exos, and HSTP1-Exos on the basis of NTA. **B** TEM images of blank-exosomes (Blank-Exos), lamp2b-exosomes (Lamp2b-Exos), and HSTP1-exosomes (HSTP1-Exos). **C** Western blot analysis of huc-MSCs and exosomes by CD6, CD63, and TSG101

We observed exosome morphology using TEM. Exosomes showed cup-shaped structures that were limited by a lipid bilayer (Fig. 5A). NTA analysis showed examined that the exosomes mainly fall within the range of 100–200 nm (Fig. 5B), which is the typical size for exosomes [25]. Western blot analysis confirmed that the huc-MSC-derived exosomes expressed and were enriched for, the known exosomal markers CD9, CD63, and TSG101(Fig. 5C). Those data indicated that genetic modification did not affect the physical properties of exosomes.

### In vitro targeting of HSTP1-Exos

**Fig. 6:**
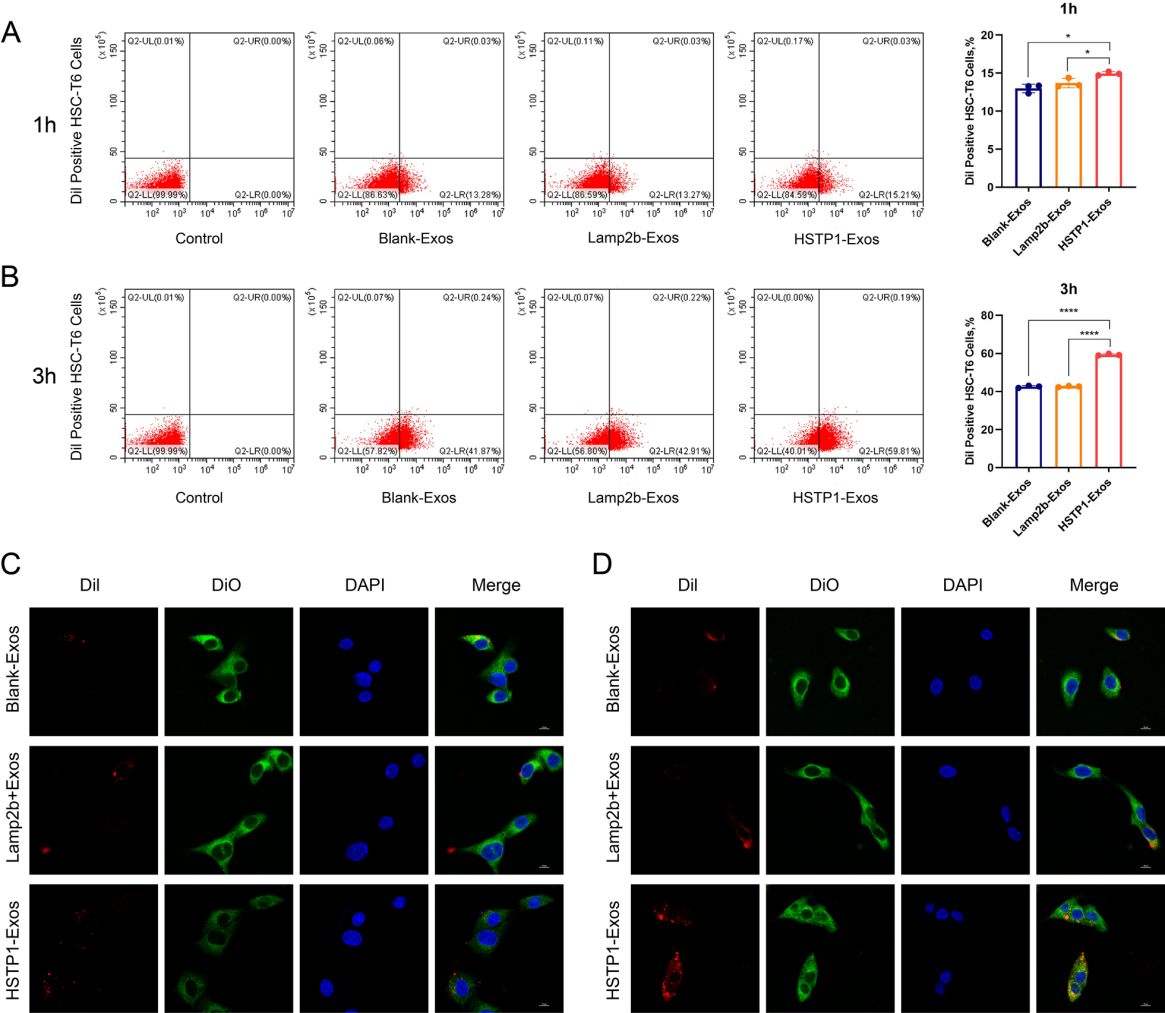
Binding of HSTP1-Exos to HSC-T6 cells in vitro. **A**, **B** Flow cytometric analysis of Blank-Exos, Lamp2b-Exos, and HSTP1-Exos labeled with Dil binding to HSC-T6 cells. The percentages represent the proportion of HSC-T6 cell that had internalized Dil-labeled exosomes (Dil-positive HSC-T6 cells) at 1 h and 3 h. **C**, **D** Confocal microscopy images of colocalization of Dil-labeled Blank-Exos, Lamp2b-Exos, and HSTP1-Exos (red) and DiO labeled cell membrane (green) at 1 h and 3 h. Cell nuclei were stained with DAPI (blue). Scale bars: 10 µm. *P < 0.05, **P < 0.01, ***P < 0.001, ****P < 0.0001

To investigate whether HSTP1-Exos could more efficiently bind to HSC-T6 cells, Blank-Exos, Lamp2b-exos and HSTP1-Exos (50 µg/ml) were labeled with Dil and cultured with HSC-T6 cells. Flow cytometry demonstrated that HSTP1-Exos bound to HSC-T6 cells more efficiently than Blank-Exos and Lamp2b-exos (14.94 ± 0.25% versus 12.99 ± 0.56% and 13.68 ± 0.61%) at 1 h, (59.3 ± 0.45% versus 42.50 ± 0.58% and 42.62 ± 0.30%) and at 3 h (Fig. 6A, B). To confirm whether HSTP1-Exos fused with HSC-T6 cells, we labeled exosomes with Dil and HSC-T6 cells with DiO. The labeled exosomes were incubated with the labeled HSC-T6 cells for 1 or 3 h at 37 ℃ before confocal laser-scanning microscopy observation. We found there were low levels of interaction in the Blank-Exos and Lamp2b-Exos up to 3 h. In contrast, merging of the red and green fluorescence on the cell surface was apparent within 1 h and increased up to 3 h (Fig. 6C, D). These findings confirmed the targeting ability of HSTP1-Exos to HSC-T6 cells.

### Anti-fibrosis effect of HSTP1-Exos in vitro

**Fig. 7:**
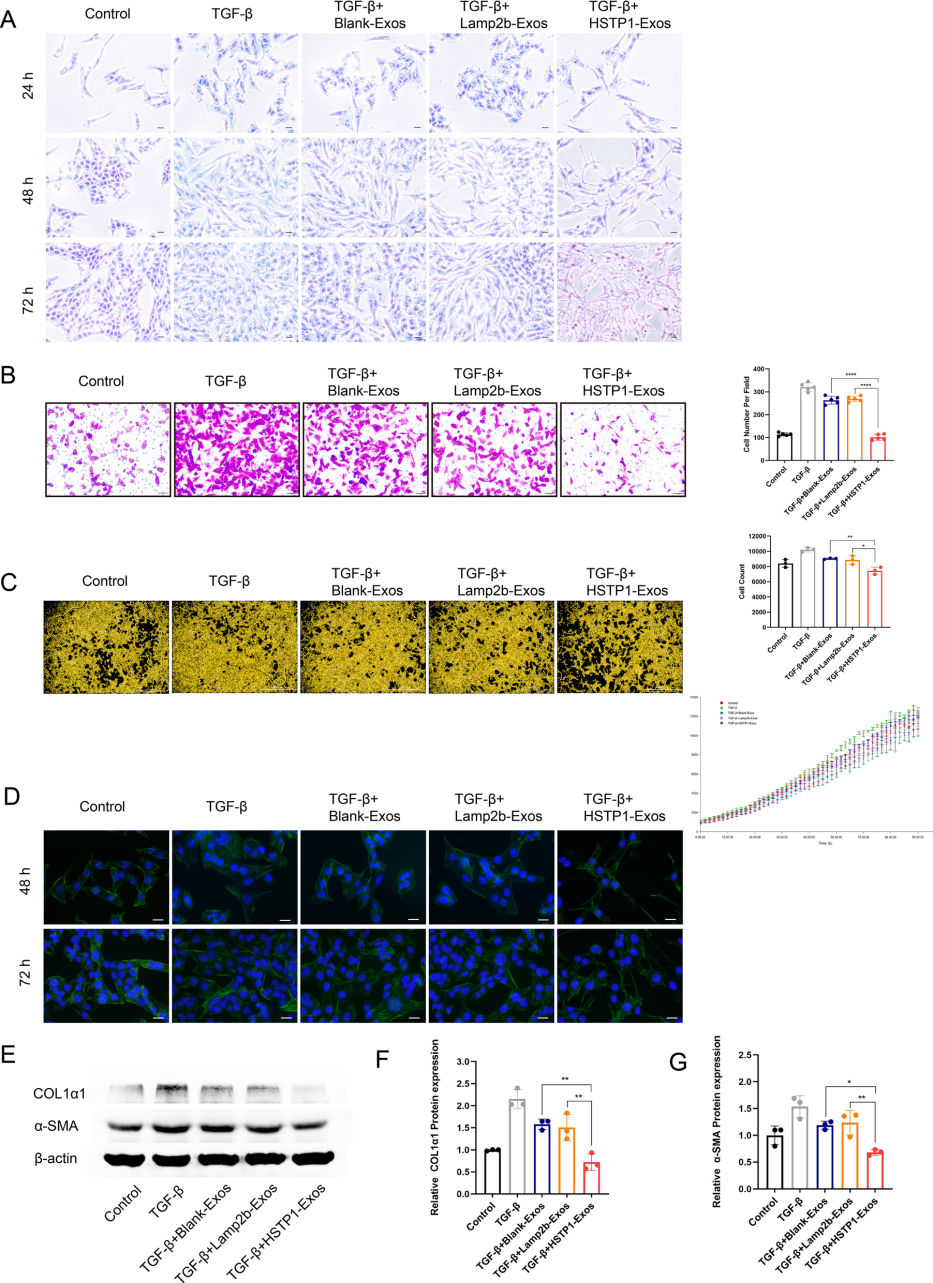
Anti-fibrosis effect of HSTP1-Exos in vitro. **A** Visualization of lipid droplets by Oil Red staining in control, TGF-β, Blank-Exos, Lamp2b-Exos, and HSTP1-Exos-treated HSC-T6 cells at 24 h, 48 h, and 72 h. Scale bars: 20 µm. **B** The effect of exosomes on the HSC-T6 cells migration ability. The bar graph showed the results of analysis of the selected fields. **C** The effect of exosomes on the HSC-T6 cells proliferation. The bar graph depicted the cell count in each group at 72 h. The line chart showed the growth curve in each group. Scale bars: 1000 µm. **D** α-SMA immunocytofluorescence (green) in control, TGF-β, Blank-Exos, Lamp2b-Exos, and HSTP1-Exos-treated HSC-T6 cells at 48 h and 72 h. DAPI-stained nuclei (blue). Scale bars: 20 µm. **E**–**G** Western blot and relative protein expression qualification for the fibrotic proteins in HSC-T6 cells. *P < 0.05, **P < 0.01, ***P < 0.001, ****P < 0.0001

To detect the anti-fibrosis function of HSTP1-Exos, we treated HSC-T6 cells with different exosomes. There were homogeneous populations of cells containing lipid droplets with any treatment. As shown by phase-contrast light microscopy, these further activated cells progressively lost their cytoplasmic lipid droplets with TGF-β1 introduction. Notably, HSTP1-Exos treatment apparently prevented the further activation of HSC-T6 cells with more abundant LDs in the cells, such as quiescent cells, within 24 h, which increased overtime (up to 72 h). There were relatively fewer LDs in the Blank-Exos- and Lamp2b-Exos-treated groups (Fig. 7A). Transwell assays indicated that TGF-β1 prominently increased the migration of HSC-T6 cells in the TGF-β1 group. Compared with the Blank-Exos and Lamp2b-Exos, HSTP1-Exos notably inhibited HSC-T6 cell migration (Fig. 7B). Moreover, live-cell imaging showed that exosomes reduced the proliferation of TGF-β1-treated HSC-T6 cells. The effect of HSTP1-Exos was more significant (Fig. 7C). We observed the α-SMA skeleton of cells by fluorescence microscopy. The cells treated with TGF-β1 were enlarged in size and more myofibroblast-like, and possessed an extended α-SMA pattern with many filopodia. In sharp contrast, the cells treated with HSTP1-Exos displayed thin, elongated processes that extended radially from the cell body. Compared with that of the TGF-β1 group, the cells morphology of the Blank-Exos- and Lamp2b-Exos-treated groups showed little change (Fig. 7D). In addition, the expression of α-SMA and COL 1α1, the typical markers of liver fibrosis, was markedly decreased in the HSTP1-Exos-treated group compared with the Blank-Exos- and Lamp2b-Exos-treated groups (Fig. 7E–G). These results indicated that modification of HSTP1 improved the delivery efficiency of exosomes and enhanced the ability of exosomes to promote reversion of aHSCs to the quiescent phenotype.

### In vivo targeting of HSTP1-Exos

**Fig. 8:**
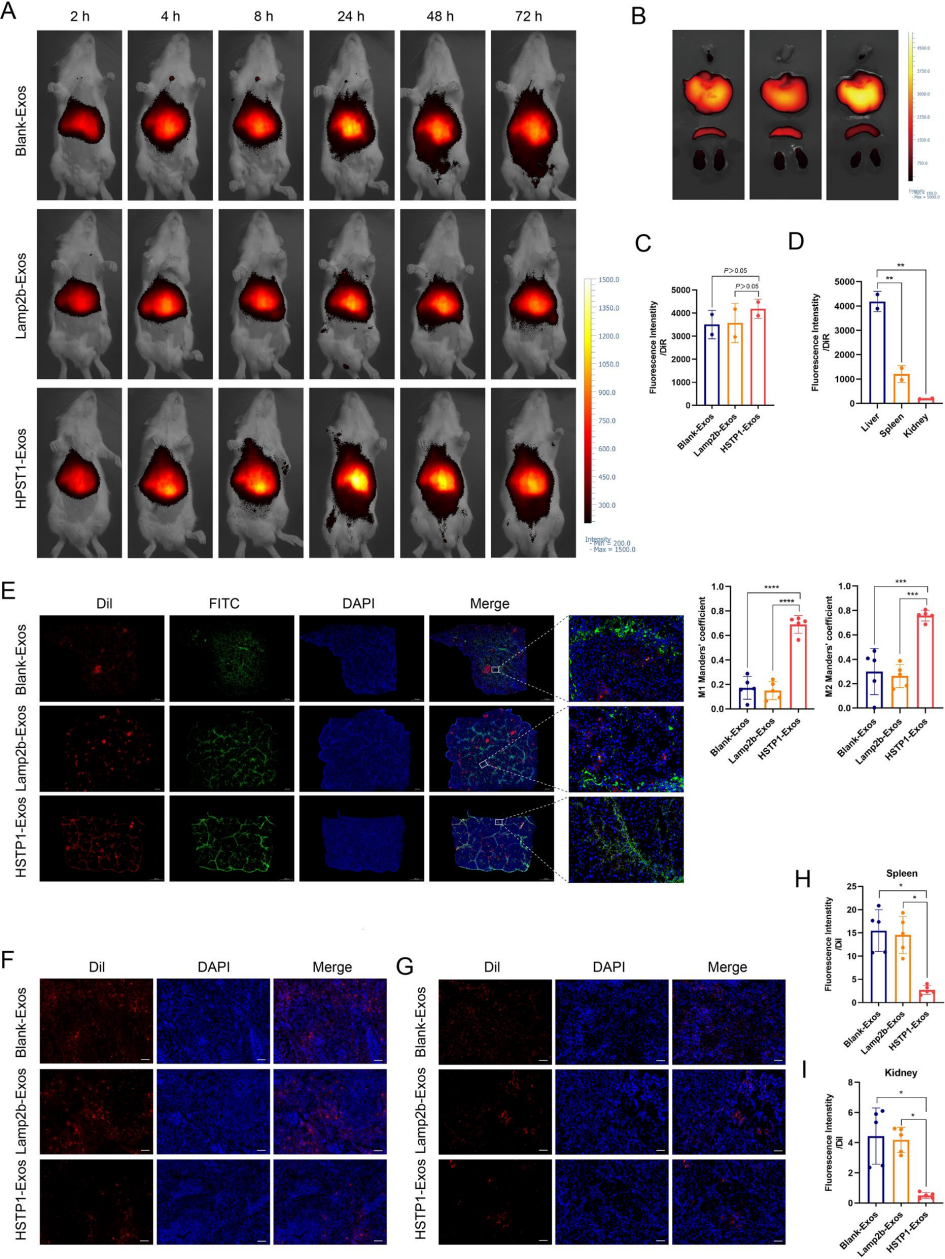
In vivo aHSCs targeting ability of the HSTP1-Exos. **A** In vivo tracking of DiR-labeled Blank-Exos, Lamp2b-Exos, and HSTP1-Exos at 2, 4, 8, 24, 48, and 72 h. **B** *Ex* fluorescence imaging of major organs from rat models of liver fibrosis 72 h after intravenous injection with DiR-labeled Blank-Exos, Lamp2b-Exos, and HSTP1-Exos. **C** Mean fluorescence intensity from the fibrotic liver quantified using a vivo smart imaging system. **D** Quantitative fluorescence intensity of liver, spleen, and kidney from the HSTP1-Exos group. **E** Confocal laser-scanning microscopy images of the cellular localization of the Dil-labeled Blank-Exos, Lamp2b-Exos, and HSTP1-Exos (red) in fibrotic liver tissues. The aHSCs were stained with anti-α-SMA antibody (green). Scale bar: 1000 µm. The bar graph depicts the Manders’ coefficients M1 and M2 in colocalization. **F**, **G** Representative images of recruitment of Dil-labeled Blank-Exos, Lamp2b-Exos, and HSTP1-Exos (red) in spleens and kidneys. **H**, **I** Quantitative fluorescence intensity of Dil-labeled Blank-Exos, Lamp2b-Exos, and HSTP1-Exos in the spleen and kidney. Scale bar: 100 µm. Cell nuclei were stained with DAPI. *P < 0.05, **P < 0.01, ***P < 0.001, ****P < 0.0001

Having shown the capacity of HSTP1-Exos for efficient targeted delivery to HSC-T6 cells, we performed experiments to assess the potential of HSTP1-Exos to specifically deliver to aHSCs in vivo. To evaluate biodistribution, we labeled Blank-Exos, Lamp2b-Exos, and HSTP1-Exos with DiR and intravenously injected them into fibrotic model rats. The imaging was performed using a VISQUE in vivo smart imaging system. The rats were imaged at 2, 4, 8, 24, 48, and 72 h after injection. The fluorescence signals from Blank-Exos, Lamp2b-Exos, and HSTP1-Exos were detected in the abdominal liver area, and peaked at 24 h after injection of exosomes. Then, the signal began to decline (Fig. 8A). For further analysis of the targeting ability of HSTP1-Exos, the rats were euthanized, and organs were isolated after 72 h. Ex vivo fluorescence imaging showed stronger fluorescence signals in the livers of the HSTP1-Exos group than in the livers of the Blank-Exos, and Lamp2b-Exos groups, although the data wasn’t statistically significant (Fig. 8B, C). There was relatively weak detection of DiR in the spleen and kidney, but robust fluorescence signal in the liver in the HSTP1-Exos group (Fig. 8D). To further investigate the cellular localization of HSTP1-Exos in fibrotic liver tissues, we injected Dil-labeled exosomes into rats. After 24 h of injection, the livers, spleens, and kidneys were removed for cryo sections. α-SMA was labeled by immunofluorescence and examined under a microscope. Strong-localization between HSTP1-Exos and α-SMA was found in the liver tissues. In contrast, Blank-Exos or Lamp2b-Exos and α-SMA overlapped very little (Fig. 8E). Moreover, compared with that in the Blank-Exos and Lamp2b-Exos groups, less Dil-labeled HSTP1-Exos accumulated in the spleen and kidney tissues (Fig. 8F–I). In fact, it is well documented that HSTP1-Exos can be specifically delivered to aHSCs in fibrotic livers.

### Anti-fibrosis effect of HSTP1-Exos in vivo

**Fig. 9:**
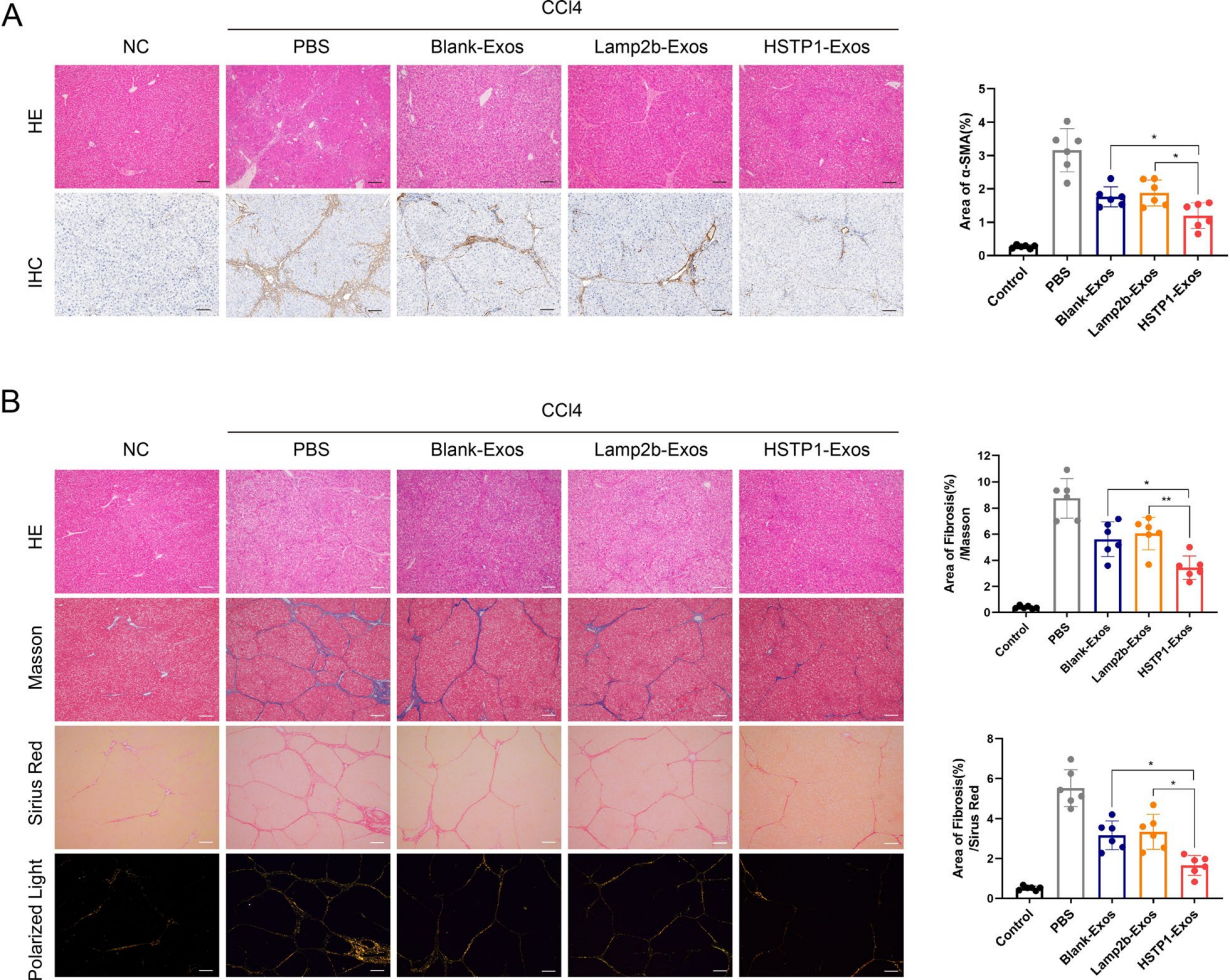
Anti-fibrosis effect of HSTP1-Exos in vivo. **A** HE staining and immunohistochemistry of α-SMA of liver tissues in NC, PBS, Blank-Exos, Lamp2b-Exos, and HSTP1-Exos groups. The bar graph depicts the quantification of α-SMA staining. **B** Histology was analyzed by HE staining, Masson’s trichrome staining, Sirius Red staining, and polarized light images of Sirius Red staining with the quantification of Masson’s trichrome staining, and Sirius Red staining. Scale bar: 100 µm. Cell nuclei were stained with haematoxylin. *P < 0.05, **P < 0.01, ***P < 0.001, ****P < 0.0001

To confirm the therapeutic potential of targeting exosomes, we injected exosomes via the tail vein twice a week for 6 weeks. HE staining showed that the CCl_4_-treated rats showed intense chronic inflammatory infiltrate in the portal area, destruction of the hepatic lobule structure and increased cell space compared to the rats in the control group. α-SMA was evaluated by immunohistochemical staining. Compared with the control, CCl_4_ significantly increased the number of α-SMA-positive cells in fibrotic liver tissues. The above liver injuries were alleviated after treatment with exosomes, and the positive areas were smaller in the HSTP1-Exos group than in the Blank-Exos and Lamp2b-Exos groups (Fig. 9A). The Masson’s trichrome staining and Sirius Red staining results were consistent with our α-SMA immunohistochemical staining, and the liver tissues from the HSTP1-Exos group showed remarkably less collagen deposition than those from the Blank-Exos and Lamp2b-Exos groups (Fig. 9B). Histologic analyses showed that modification of HSTP1 could enhance the therapeutic effects of the huc-MSC-derived exosomes on liver fibrosis, because of the effective accumulation of exosomes.

### Regulation of HSTP1-exos on macrophage

**Fig. 10:**
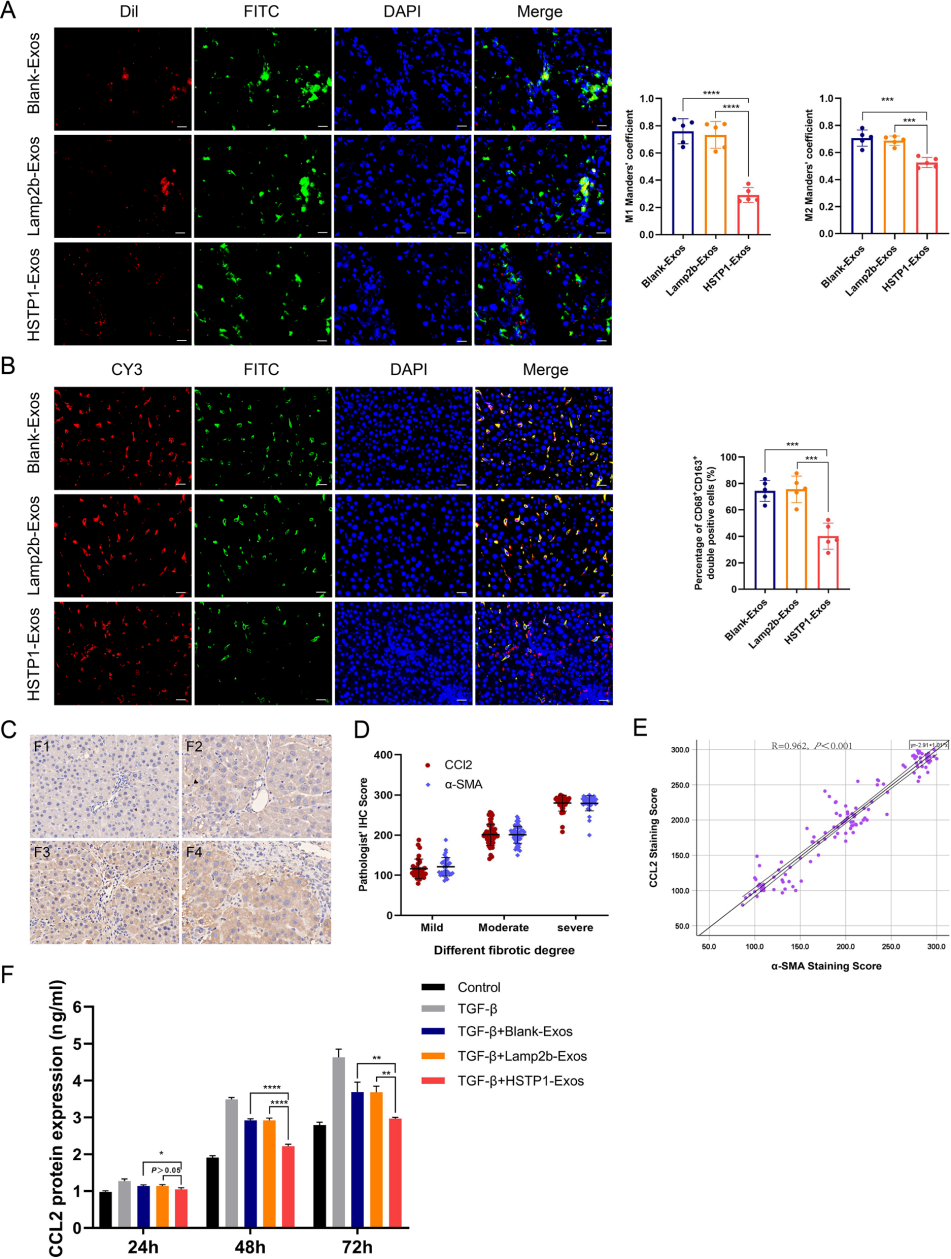
Regulation of HSTP1-Exos via inhibiting CCL2 secretion from aHSCs. **A** Cellular uptake of the Dil-labeled Blank-Exos, Lamp2b-Exos, and HSTP1-Exos (red) into FITC-labeled CD68^+^ macrophage (green) in fibrotic liver tissues. The bar graph depicts the Manders’ coefficients M1 and M2 in colocalization. **B** Immunofluorescence double staining of liver tissue with CY3-labeled CD68 and FITC-labeled CD163. The bar graph depicts the percentage of CD68^+^CD163^+^ double positive macrophages to CD68 + positive macrophages in each group. **C** Representative images of immunohistochemistry staining of liver tissues antibody of CCL2. The degree of liver fibrosis was assessed by the Metavir score system (F0, F1, F2, F3, and F4). **D** The correlation between α-SMA and CCL2 under different fibrotic degree. F0 and F1 were defined as mild fibrosis, F2 and F3 as moderative fibrosis, and F4 as severe fibrosis. Protein expression was quantified based on the evaluation of staining using semiquantitative Histoscore. **E** The scatter plot of a correlation between aHSCs (α-SMA) IHC staining score and CCL2 score. **F** Elisa analysis of CCL2 protein expression levels in exosomes treated HSC-T6 cells. Scale bar: 20 µm. *P < 0.05, **P < 0.01, ***P < 0.001, ****P < 0.0001

To evaluate the uptake of different exosomes by macrophages, we made cryo sections of exosome-injected liver tissue. Visual scoring of Manders’ coefficients of the tissue samples showed limited colocalization of Dil-labeled HSTP1-Exos and FITC-labeled CD68 in HSTP1-Exos group, which were commonly characterized by the strong overlap of the two fluorescence signals in both Blank-Exos group and Lamp2b-Exos group (Fig. 10A). To determine the changes in macrophage phenotype after treatment of exosomes, we use immunofluorescence to label CD68 and CD163 in paraffin sections of liver fibrosis. As shown, there was less detection of CD68^+^CD163^+^ double positive macrophages in HSTP1 group than that in the Blank-Exos group and Lamp2b-Exos group (Fig. 10B). In a previous study found that aHSCs could induce infiltration and formation of M2 macrophage (CD163^+^) via CCL2/CCR2 pathway. Herein, we found the expression of CCL2 increased with liver fibrosis degree (Fig. 10C). Further statistical analyses were also performed for possible correlation among α-SMA and CCL2. The correlation between α-SMA and CCL2 were explored under different fibrotic degree and by scatter plot (Fig. 10D, E), which suggested a correlation between the aHSCs (α-SMA) and CCL2 (R = 0.962, *P* < 0.001). Additionally, HSC-T6 cells were treated with different exosomes and supernatants were collected for measuring the concentration of CCL2. ELISA showed that exosomes could effectively inhibit TGF-β-induced CCL2 expression, and compared with Blank-Exos and Lamp2b-Exos, the effect of HSTP1-Exos was more obvious. HSTP1-Exos totally abolished the up-regulation of CCL2 after being treated by TGF-β (Fig. 10F). These results indicated that HSTP1-Exos could regulate M2 macrophage (CD163^+^) polarization by inhibiting CCL2 secretion from aHSCs.

## Discussion

Liver fibrosis is a common progressive pathological process of different chronic liver injuries [26]. The aHSCs play a central role in the development of liver fibrosis and give rise to approximately 90% of myofibroblasts in a fibrotic animal model [27]. However, effective therapeutics to stop or reverse liver fibrosis have not emerged, because these potential agents cannot specifically target aHSCs or are frequently toxic to parenchymal cells [5]. The discovery of ligands that selectively bind to a specific target plays a crucial role in clinically relevant diagnostics and therapeutics. Phage display has been used for performing high-throughput screening and identifying functional peptide ligands for various targets, including PD-L1 [28], tumor stromal cells [29], and ischemic myocardial tissues [30]. These ligands offer targeting fragments for the construction of efficient diagnostic and therapeutic platforms [31]. In this study, we identified the targeting peptide of aHSCs, HSTP1, by biopanning with a phage display peptide library. To prevent effects on parenchymal cells, we specifically selected BRL-3A cells as the negative control cells. Notably, HSTP1 not only stably binds to HSC-T6 cells, but also shows high specificity for aHSCs in pathological sections of fibrotic liver, similar to the effect of anti-α-SMA antibody. Compared with antibodies, peptides appear to cost less to produce and perform quality control during peptide synthesis. HSTP1 might be a promising alternative tool for the pathological diagnosis of liver fibrosis. Moreover, HSTP1 provides a reliable ligand for the targeted delivery of drug molecules, oligonucleotides, liposomes, and exosomes.

Accumulating evidences has shown that exosomes are emerging as promising natural nanovectors, given their unique properties, including low immunogenicity, high biocompatibility, and low toxicity [32, 33]. Moreover, MSC-derived exosomes are believed to be a potential therapeutic tool for liver fibrosis [34]. In vivo, in an animal model, huc-MSC-derived exosomes reduced the surface fibrous capsules and softened their textures by inhibiting EMT [35]. This change could be associated with inhibition of the profibrogenic response of HSCs and decreased the expression of the profibrogenic markers α-SMA, and collagen I stimulated by TGF-β1 in vitro [13]. Concretely speaking, MSCs could release exosomes containing miR-125b to impeding the activation of Hedgehog-responsive HSCs via the inhibition of Smo expression [36]. Exosomes produced by adipose tissue-derived MSCs expressing miR-122 could also reducing the proliferation and activation of the HSCs [15]. However, the clearance rate of exogenous exosomes after systemic injection showed no differences from that of liposomes due to poor targeting of unmodified exosomes, which are mainly taken up by the mononuclear phagocyte system [20, 37]. The intravenous delivery of exosomes into aHSCs while preventing unintended delivery remains a challenge. In the present study, we first displayed a targeting peptide was displayed on the surface of huc-MSC-derived exosomes to improve the specificity and efficiency of delivery to aHSCs. Additionally, we added the glycosylation motif GNSTM to the N-terminus of HSTP1 to protect the targeting peptide from degradation and increase the total amount of fusion protein present in cells and exosomes.

Large numbers of qHSCs reside in normal livers, suggesting that these cells play important homeostatic roles [38]. When the liver is subjected to injuries, HSCs gradually lose vitamin A, migrate to the injury sites, and transdifferentiate into myofibroblast-cells expressing α-SMA and collagen [27]. Activation and migration of HSCs are critical in pathogenesis of liver fibrosis [39]. HSCs possess remarkable plasticity, and aHSCs (myofibroblasts) revert to a quiescent state, termed “deactivation”, with reduced α-SMA and I collagen expression which is followed by the regression of liver fibrosis [40]. The reversion of aHSCs back to a more quiescent phenotype has attracted increased attention as an ideal therapeutic option for liver fibrosis. When autophagic flux in cells is enhanced by TGF-β1, LDs in HSCs are recognized through autophagy and are subjected to lysosomal degradation. Free fatty acids released by LD degradation are thought to be the energy sources for the fibrogenic response in HSCs. Blocking autophagy promoted lipid accumulation in HSCs with a quiescent phenotype, and attenuated liver fibrosis in vivo [41]. At present, no study has confirmed that huc-MSC-derived exosomes promote the deactivation of fibrogenic HSCs, which is related to autophagic regulation, but apparently more LDs accumulated in the cytoplasm of HSC-T6 cells in the HSTP1-Exos treated group. In this study, transdifferentiation of aHSCs to a relatively quiescent phenotype not only involved a decrease in migration proliferation but also extensive remodeling of the cytoskeleton. Immortalized HSC-T6 cells do exhibit an activated phenotype. When further activated by TGF-β1, HSC-T6 cells were enlarged in size and possessed an extended α-SMA cytoskeleton. Conversely, the cells treated with HSTP1-Exos were small and displayed thin, elongated extensions. In vitro experiments showed that HSTP1-Exos could be internalized by HSC-T6 cells more efficiently than Blank-Exos and Lamp2b-Exos, and improved the therapeutic effect on transdifferentiation of HSC-T6 cells induced by TGF-β1.

Current targeted HSC vectors have a low delivery efficiency and are frequently toxic to parenchymal cells and lack specificity [42, 43]. Based on our data, intravenously administered HSTP1-Exos in an acceptable route of administration, significantly accumulated in the aHSC region. This phenomenon effectively suppressed the proliferation of aHSCs and collagen deposition; it benefited from the targeting of HSTP1 and the ability of exosomes to penetrate physiological barriers that were impermeable to synthetic nanoparticles [44]. Indeed, in our work, a low dosage of HSTP1-modified exosomes was able to reduce CCl_4_-induced liver fibrosis in vivo.

In this study, we made a preliminary exploration on immunomodulatory function of MSC-derive exosomes. Nanomedicine has advantages over conventional therapeutics such as multi-functionality, efficient drug delivery, and controlled release of the drug cargos. Compared to conventional small molecule-based therapeutics, synthetic nanoparticles are easily phagocytosed up by the reticuloendothelial system [45, 46]. Despite multiple advantages over existing systems, such as less immunogenicity and higher affinity, exosomes suffer from the drawback of endocytosis by the mononuclear phagocyte system [19]. Consistent with previous studies, exosomes without HSTP1 modification were more taken up by macrophages in livers. MSC-derived exosomes play a central role in inducing anti-inflammatory M2 macrophage polarization. However, M2 macrophages promote tissue remodeling and the progression. In contrast, M1 phenotype is characterized by high level expression of matrix metalloproteinase-2, -9, -13, leading to anti-fibrotic activity in the resolution of hepatic fibrosis [47]. In a previous study found that aHSCs could induce infiltration and formation of M2 macrophage (CD163^+^) via CCL2/CCR2 pathway [48]. HSTP1-modified exosomes not only reduced the chance of being taken up by macrophage, but also enhanced the inhibition of CCL2 secretion from aHSCs. This may provide some clues and also be one of the reasons why HSTP1-modified exosomes were effective in treating liver fibrosis in vivo experiments.

Although this study demonstrated that HSTP1 modified exosomes showed superiority in the treatment of liver fibrosis compared to the natural exosomes, there are still some limitations. First, we do not explore the target protein receptors for HSTP1. Of course, the screening procedure of phages may involve a specified target molecule with prior knowledge of the molecular structure or whole cells, tumor or tissue can be directly used as target without any prior knowledge of specific binding site [49]. Secondly, we only confirm the functional roles of HSTP1 modified exosomes, and do not identify the clear mechanism of huc-MSC-exosomes alleviating HSC activation. Exosomes can contain varied genetic information derived from the donor cell, and involve in multiple cellular activities, including antigen presentation, signal transduction, and immune response. When exosomes reach a target cell, they can affect the physiological and pathological state of recipient cells through transferring their contents, including protein molecules, lipids, and nucleic acids [50]. To improve the therapeutic effect of fibrotic hepatic hepatic diseases, it would be necessary to identify the exact intercellular messengers in further studies.

In conclusion, these findings suggest that HSTP1 is a reliable targeting peptide that can specifically bind to aHSCs as well as a promising molecular imaging probe for the pathological diagnosis of liver fibrosis. In this study, we creatively displayed targeting peptides of aHSCs on the surface of MSC-derived exosomes for liver fibrosis therapy. The modification of HSTP1 realizes the precise treatment of nanomedicine for a single type of cell in complex liver tissue, and improves the ability of exosomes to reverse liver fibrosis. We provide a novel approach for clinical treatment of liver fibrosis.

## Methods

### Cell culture

Rat hepatic stellate cells (HSC-T6) (Procell Life Science & technology Co., Ltd, Wuhan, China), rat liver cells (BRL-3A), rat kidney epithelial-like cells (NRK-52E) and rat cardiomyoblast cells (H9C2) (Chinese Academy of Science, Shanghai, China) were maintained in DMEM (Biological Industries, Israel) containing 10% fetal bovine serum (FBS) (HyClone, USA) in an incubator with 5% CO_2_ at 37 °C.

### Screening the targeting peptide of HSC-T6 cells

Phage display biopanning was performed according to a previous description, with certain modifications [51]. HSC-T6 cells were selected as the positive target cells, and BRL-3A cells were selected as the negative bound cells for whole-cell subtractive screening from the Ph.D. C7C™ Phage Display Library (New England Biolabs, USA). A total of 4 rounds of biopanning were performed, 2 × 10^11^ plaque-forming units (pfu) of collected phage were used for each round, and Tween 20 was increased in a stepwise manner to 0.2%. Twenty phage clones were randomly selected, and amplified. Sanger sequencing was performed by GENEWIZ, Inc., (Suzhou, China). The data were analyzed using Chromas 2.6.5 (South Brisbane, Australia).

### Enzyme linked immunosorbent assay (ELISA)

Cell-based ELISA, HSC-T6, BRL-3A, NRK-52E and H9C2 cells were plated in 96-well plates (5 × 104 cells/well) until they adhered and covered the bottom of the wells. The cells were washed three times with PBS and then fixed with 4% paraformaldehyde for 15 min. After three washes, the cells were blocked with 5% BSA for 1 h at 37 °C. Next, 2 × 10^8^ pfu of picked phages were incubated separately with each cell type for 1 h at room temperature (RT). The wells were washed 5 times, and 100 µl of horseradish peroxidase HRP-conjugated anti-M13 antibody (Abcam, UK) (1:500) was introduced into to each well for 1 h at 37 °C. After PBS washes, TMB substrate solution (Boster, China) was added to the wells and incubated for 10 min at RT. The reaction was terminated by the TMB stop solution (0.5 M H_2_SO_4_) (Boster, China). The plates were read on a full wavelength microplate reader (BioTek, Germany) at 450 nm. According to the sequencing and ELISA results, a specific peptide was identified in the HSC-T6 cell affinity clones, and designated HSTP1. A peptide with the same amino acids as HSTP1 in a randomized order as the negative control was designed as rcHSTP1. The cationic penetrating peptide RKKRRQRRR (peptide Tat) was used as the positive control. All peptides were attached to fluorescein-5-isothiocyanate (FITC) labeling and synthesized by Chinese Pepide (Hangzhou, China).

ELISA for CCL2 secreted by HSC-T6 cells, HSC-T6 cells were treated with TGF-β1 and different exosomes for 24, 48, and 72 h; then, the supernatants were centrifuged at 10,000*g* for 10 min and collected for analysis. In addition, the media from untreated were collected as the control. Commercial CCL2 ELISA kits (Elabscience, China) were used to detect the content of CCL2 in each group.

### Peptide-affinity assay

For immunocytofluorescence analysis, HSC-T6 cells, and control BRL-3A cells, and NRK-52E cells were plated in 35 mm glass bottom petri dishes (Thermo, USA) for 12 h (1 × 10^4^ cells/well). HSC-T6 cells were incubated with increasing concentrations (5, 15, 25, 50 µM) of FITC-HSTP1 for 1 h. For the examination of HSTP1 specificity, HSC-T6, BRL-3A, NRK-52E, and H9C2 cells were incubated with 15 µM FITC-HSTP1 for 1 h at RT. The cells were also incubated with 150 µM rhodamine phalloidin (Solarbio, China) for 30 min, and counterstained with DAPI (2 µg/ml) for 5 min. The cells were observed by laser scanning confocal fluorescence microscopy (Olympus, Japan).

For flow cytometry analysis, HSC-T6 cells (1 × 10^6^) were harvested and resuspended to a single cell suspension, and then divided into quarters followed by incubation with 15 µM FITC-labeled peptides for 30 min in the dark. HSC-T6 cell affinity properties of peptides were measured using flow cytometry (Beckman Coulter, USA). BRL-3A cells were treated in the same manner.

For immunofluorescencehistochemistry analysis, paraformaldehyde-fixed rat livers were stained with FITC-HSTP1, FITC-rcHSTP1 and anti-α-SMA antibodies (SAB, USA), followed by CY3-conjugated secondary antibodies. Tissue slides were imaged by fluorescence microscopy (Olympus, Japan).

### Isolation and culture of huc-MSCs

Huc-MSCs were obtained from fresh umbilical cord tissue of healthy donors using the explant method. All procedures were approved by the ethics committee. The two arteries and one vein in the umbilical cord were removed using a scalpel. Wharton’s jelly was minced into 2 mm^3^ blocks. After culture for 3 h at 37 °C, DMEM/F12 (Biological Industries, Israel) containing 15% FBS was added to T75 flasks. Approximately 7 days later, the cells gradually migrated out. Huc-MSCs were incubated with FITC-labeled primary antibodies raised against CD45 and CD73, and APC-labeled primary antibodies raised against CD105 and HLA-DR (Biolegend, USA) for 30 min at RT. After washing and resuspension in PBS, the samples were analyzed by a flow cytometer (BD, USA).

### Plasmid construct and virus package

The fusion gene sequence of Lamp2b and HSTP1 (Lamp2b + HSTP1) was synthesized and purified by Shanghai GeneChem Co., Ltd. (Shanghai, China). After being digested by Agel restriction enzymes, the lentivirus-based vectors Ubi-MCS-SV40-EGFP-IRES-puromycin and Lamp2b + HSTP1 were expanded by polymerase chain reaction (PCR), and products were run on a gel for purification. Then, the cDNA of Lamp2b + HSTP1 was added to the vector via Exnase ™ II (Clontech, USA) for 30 min at 37 ℃, followed by driving the recombinant plasmid vector into competent cells. An individual colony was picked out from agarose plates. Sequencing was conducted after bacterial explanation. Lentivirus was generated by transfection of HEK 293 T cells with the gene-transfer-plasmids, the packaging plasmid helper 1.0 and the envelope-plasmid helper 2.0. After forty-eight hours, the lentivirus suspension was harvested, and concentrated by ultracentrifugation at 25,000 r for 2 h at 4 °C.

### Transmission electron microscopy (TEM) and nanoparticle tracking analysis (NTA)

The supernatants were collected to isolate exosomes via ultracentrifugation according to a previous study [52]. The supernatants were centrifuged at 800 g for 15 min followed by 10,000 rpm for 30 min at 4 °C to remove cells and debris and then ultrafilter and concentrated at 2000 rpm for 20 min using ultrafiltration centrifugal tube (Millipore, USA). Concentrated supernatants were centrifuged at 140,000*g* for 90 min at 4 °C in a Type Ti100 rotor using an XL-100K ultracentrifuge (Beckman). After resuspension in PBS, the exosome pellet was ultracentrifuged again for 90 min at 140,000*g*. Finally, the exosomes were resuspended in PBS, filtered using a 0.22-μm filter (Millipore, USA), and analyzed with a Enhance BCA Protein Assay kit (Beyotime, China). Approximately 6 mg of exosomes are obtained per 500 ml of supernatant. The purified exosomes were resuspended in PBS (200 µl) and further diluted by 1- to 10- hundred folds for analysis. The samples were fixed with 2.5% glutaraldehyde overnight at 4 °C. Ten microliters of the mixture were applied to copper grids and stained with 1% phosphotungstic acid for 1 min. The dried grid was observed by a Tecnai G2 TEM (FEI, USA). NTA was conducted using a Zeta View system (NanoSight, UK) to automatically track the Brownian motion and size distribution data of exosomes in real time.

### Exosome uptake analysis

For investigation of the uptake of HSTP1-Exos by HSC-T6 cells, Blank-Exos, Lamp2b-Exos, and HSTP1-Exos were labeled with Dil (Thermo Fisher Scientific, USA) and cocultured with HSC-T6 cells. After 1 h and 3 h, the percentage of uptake of exosomes by HSC-T6 cells was examined by flow cytometry (Beckman Coulter, USA). Then, we labeled the HSC-T6 cells with DiO (Thermo Fisher Scientific, USA). The labeled exosomes were cocultured with HSC-T6 cells for 1 and 3 h. Images of exosomes and HSC-T6 cells were observed under confocal fluorescence microscopy (Nikon, Japan).

### LDs staining

HSC-T6 cells were divided into five groups: the normal control group, TGF-β group (cells were treated with 10 ng/ml TGF-β1), TGF-β + Blank-Exos group (cells were treated with 10 ng/ml TGF-β1 plus 50 µg/ml blank-exosomes), TGF-β + Lamp2b-Exos group (cells were treated with 10 ng/ml TGF-β1 plus 50 µg/ml lamp2b-exosomes), and TGF-β + HSTP1-Exos group (cells were treated with 10 ng/ml TGF-β1 plus 50 µg/ml HSTP1-exosomes). Cells were plated in 6-well plates for 12 h, followed by administration of the corresponding drugs for 24, 48, and 72 h. HSC-T6 cells were fixed for 15 min with 4% paraformaldehyde on the indicated culture day. Then, the cells were incubated with ORO (Sigma, Germany) working stain for 30 min, followed by 15 s of rinsing in 60% isopropanol. The nuclei were counterstained with hematoxylin for less than 1 min before phase contrast microscope observation (Olympus, Japan).

### Transwell assay and live-cell imaging

HSC-T6 cells were also divided into the control group, TGF-β group, TGF-β + Blank-Exos group, TGF-β + Lamp2b-Exos group, and TGF-β + HSTP1-Exos group, and treated with the corresponding drugs for 48 h. Each group of cells was harvested, and 2 × 10^4^ cells in 200 µl of nonserum medium were placed in the upper chamber of an insert. The lower chamber was filled with 700 µl of DMEM with 20% FBS. Migrated cells on the underside of the filter, were fixed and stained with 1% crystal violet for 10 min. Three randomly selected fields were photographed and counted under a phase contrast microscope (Olympus, Japan). HSC-T6 cells (3 × 10^3^ cells/well) were seeded in 96-well culture plates. After attachment, the cells were treated with TGF-β1 and exosomes. Cell count was detected using Cytation C5 (BioTek, USA).

### Immunofluorescence staining

HSC-T6 cells, divided into the control group, TGF-β group, TGF-β + Blank-Exos group, TGF-β + Lamp2b-Exos group, and TGF-β + HSTP1-Exos group, were seeded on slides in 6-well plates, followed by administration of the corresponding drugs for 48 h, and 72 h. The cells were permeabilized with 0.5% Triton X-100 for 20 min and blocked with 3% bovine serum albumin (BSA) for 1 h. Then, the cells were incubated with anti-α-SMA antibody (SAB, USA) for 16 h at 4 °C, followed by incubation with FITC-conjugated secondary antibody for 1 h at RT. The nuclei were counterstained with DAPI, and images were taken using a fluorescence microscope.

### Western blot and real-time PCR

The BCA protein assay kit was used to determine the protein concentration. Proteins were fractionated by 10% sodium dodecyl sulfate–polyacrylamide gel electrophoresis (SDS-PAGE) and transferred onto a PVDF membrane. The membrane was blocked with 3% BSA in Tris-buffered saline containing 0.05% Tween 20. Antibodies against CD9, CD63, TSG101, GAPDH (Abcam, USA), Lamp2 (Santa Cruz, USA), CoL1α1, α-SMA, and β-actin (SAB, USA) were used to quantify protein expression by Western blotting. Total RNA was extracted with TRIzol (TaKaRa, Japan). Real-time PCR was performed using an Mx3000 real-time PCR detection system (Agent, USA). The 2^−△△Ct^ method was used to calculate the relative expression.

### Animal treatment

Four-week-old male SD rats (n = 120) weighing 120–150 g were purchased from the Lanzhou Veterinary Research Institute of Chinese Academy of Agricultural Sciences (Lanzhou, China). After a week-long acclimation period, rats were randomly divided into normal control (NC) groups and experimental groups. The experimental group was subjected to intraperitoneal injections of 2 ml CCl_4_/olive oil (1:1, v/v)/kg body weight 2 times per week for up to 6 weeks to induce the liver fibrosis model. Then, the rats in the experimental group were divided into 4 groups: PBS, Blank-Exos, Lamp2b-Exos, and HSTP1-Exos groups. Rats in the PBS group were given 500 µl of PBS twice a week for 6 weeks, and rats in the Blank-Exos, Lamp2b-Exos, and HSTP1-Exos groups were given corresponding exosomes 2.5 mg/kg in 500 µl PBS twice a week for 6 weeks. Blank-Exos, Lamp2b-Exos, and HSTP1-Exos, labeled with DiR, were intravenously injected via the tail vein to determine tissue distribution via a VISQUE in vivo smart imaging system (Vieworks, Korea). Blank-Exos, Lamp2b-Exos, and HSTP1-Exos, labeled with Dil, were intravenously injected via the tail vein to determine the cell distribution via cryosections. The tissues were embedded with OCT and sliced at − 20 °C; then, liver tissue slices were immunostained with anti-α-SMA (SAB, USA), or anti-CD68 (Abcam, UK) antibodies followed by FITC-conjugated secondary antibodies (Abcam, UK). Tissue slides were imaged by fluorescence microscopy (Olympus, Japan).

At the end of the experiments, liver tissues were removed and fixed in 4% neutral formaldehyde, embedded in paraffin and sectioned. HE staining, Masson’s trichrome staining, and Sirius red staining were used for analysis of histological structure and fibrotic area, respectively. The positive area was assessed with ImageJ 1.8.0 software (National Institutes of Health, USA). CD68^+^CD163^+^ double positive macrophages were analyzed by immunofluorescence staining with anti-CD68 and anti-CD163 antibodies (Abcam, UK) followed by CY3- and FITC-labeled goat anti-rabbit IgG antibodies (Abcam, UK) respectively. For immunohistochemistry analysis, the slices were probed with primary targeted against rat α-SMA (SAB, USA) and CCL2 (Proteintech, China), and stained with 3,3’-diaminobenzidine. The degree of liver fibrosis in different rats in PBS, Blank-Exos, Lamp2b-Exos, and HSTP1-Exos groups were assessed according to the Metavir score system. Under 400-times microscope, 5 pathological areas were randomly selected. The CCL2 and α-SMA protein expression was quantified based on the semi-quantitative Histoscore, which was calculated by an assessment of both the percentage of positive cells and the intensity of staining (0, non-staining; 1, weak; 2, median; or 3, strong). This analysis was performed by two independent reviewers.

### Statistical analysis

All data are expressed as the mean ± SD. Independent sample t test was applied for comparing data between two groups, and one-way analysis of variance (ANOVA) was utilized for data comparison among multiple groups. *P*-values less than 0.05 were considered statistically significant. Statistical analyses were performed using Prism 8 software (GraphPad, USA).

## Data Availability

The datasets used and/or analysed during the current study are available from the corresponding author on reasonable request.
